# Symptoms in Swiss adolescents in relation to exposure from fixed site transmitters: a prospective cohort study

**DOI:** 10.1186/s12940-016-0158-4

**Published:** 2016-07-16

**Authors:** Anna Schoeni, Katharina Roser, Alfred Bürgi, Martin Röösli

**Affiliations:** Swiss Tropical and Public Health Institute, Socinstrasse 57, P.O. Box, CH-4002 Basel, Switzerland; University of Basel, Basel, Switzerland; ARIAS umwelt.forschung.beratung, Bern, Switzerland

**Keywords:** Geospatial propagation model, Adolescents, Symptoms, Fixed site transmitter, RF-EMF

## Abstract

**Background:**

There is public concern regarding potential health effects of radiofrequency electromagnetic fields (RF-EMF) emitted by fixed site transmitters. We therefore investigated whether self-reported general well-being in adolescents is affected by RF-EMF exposure from mobile phone base stations (downlink) and broadcast transmitters (TV and radio).

**Methods:**

In a prospective cohort study, 439 study participants aged 12-17 years, completed questionnaires about their self-reported well-being and possible confounding factors at baseline and one year later. Exposure from fixed site transmitters at home and school was calculated by using a geospatial propagation model.

Data were analysed using a mixed-logistic cross-sectional model of a combined dataset consisting of baseline and follow-up data and a longitudinal approach where we investigated whether exposure at baseline (cohort analysis) or changes in exposure between baseline and follow-up (change analysis) were related to a new onset of a symptom between baseline and follow-up. All analyses were adjusted for relevant confounders.

**Results:**

Mean exposure (median; 75^th^) for broadcast transmitters, downlink and total exposure at baseline were 1.9 μW/m^2^ (1.0 μW/m^2^; 2.8 μW/m^2^), 14.4 μW/m^2^ (3.8 μW/m^2^; 11.0 μW/m^2^) and 16.3 μW/m^2^ (5.8 μW/m^2^; 13.4 μW/m^2^), respectively. In cross-sectional analyses no associations were observed between any symptom and RF-EMF exposure from fixed site transmitters. In the cohort and change analyses only a few significant associations were observed including an increased OR for tiredness (2.94, 95%CI: 1.43 to 6.05) for participants in the top 25^th^ percentile of total RF-EMF exposure from fixed site transmitters at baseline, in comparison to participants exposed below the median and a decreased OR for exhaustibility (0.50, 95%CI: 0.27 to 0.93) for participants with an exposure increase between baseline and follow-up.

**Conclusions:**

In this cohort study, using a geospatial propagation model, RF-EMF exposure from fixed site transmitters was not consistently associated with self-reported symptoms in Swiss adolescents. The few observed associations have to be interpreted with caution and might represent chance findings.

**Electronic supplementary material:**

The online version of this article (doi:10.1186/s12940-016-0158-4) contains supplementary material, which is available to authorized users.

## Background

Number of sources emitting radio-frequency electromagnetic fields (RF-EMF) such as base stations, mobile and cordless phones, broadcast transmitters and WLAN have substantially increased in the everyday environment during the last few decades. This increase has been accompanied by a growing public concern that RF-EMF may have an effect on human health; especially on non-specific symptoms like headache or sleep disturbances. The majority of RF-EMF research so far has focused on the exposure from mobile phones whereas the exposure from broadcast transmitters (TV and radio) and base stations has received less attention. This might be due to the relative low induced exposure levels from broadcast transmitters and base stations compared to the exposure that is induced by mobile phones and other wireless communication devices operating close to the body.

According to a systematic review [1] where human experimental and epidemiological studies until March 2009 were included, not one single symptom or symptom pattern was consistently related to exposure from mobile phone base stations. In the epidemiological studies, a tendency towards increased symptom reports was observed in studies using subjective exposure surrogates (e.g., self-estimated distance to closest mobile phone base station), while no effects could be shown in studies with objective exposure surrogates. However, studies in children and adolescents were scarce. The only experimental study investigating effects of mobile phone base station exposure on health symptoms that included adolescents was from Riddervold et al. [2]. They observed a larger change in headache score after UMTS exposure than after sham exposure when the data from 40 adults and 40 adolescents were pooled. However, this change was due to a lower headache baseline score before exposure rather than to a higher score after exposure. In an epidemiological study (MobilEe-study), using 24 h personal measurements for assessing RF-EMF exposure no consistent associations between measured exposure and acute symptoms in children and adolescents were seen [3]. Some associations reaching statistical significance were not consistent over two time points (morning and afternoon) and the authors hypothesized that the observed associations are due to chance because of multiple testing. Additionally, they did not only consider exposure from fixed site transmitters because the dosimeter was limited to differentiate between uplink (mobile phone handsets) and downlink bands. In the same study they investigated associations between measured exposure and chronic symptoms [4]. They did not find any association between individual personal RF-EMF exposure and chronic well-being although measured RF-EMF exposure in the highest quartile was associated to overall behavioural problems for adolescents but not for children [5].

We aimed thus to investigate whether self-reported general well-being in Swiss adolescents is affected by RF-EMF exposure from mobile phone base stations and broadcast transmitters using a geospatial propagation model.

## Methods

### Study population

For the present study, as part of the HERMES (Health Effects Related to Mobile phonE use in adolescentS) study, adolescents from 7th, 8th and 9th grade in schools from rural and urban areas in Central Switzerland were recruited. The baseline investigation took place between June 2012 and February 2013. During a school visit the adolescents filled in a questionnaire with questions on non-specific symptoms of ill health, socio demographics, and other relevant covariables. This information was complemented by a parental questionnaire with additional items such as house characteristics. Parents were asked to fill out the questionnaire and send it back directly. Teachers were asked to fill out a questionnaire with questions on school building characteristics and floor location of the class room. This procedure was repeated one year later with the same study participants and the same study managers.

Ethical approval for the conduct of the study was received from the ethical committee of Lucerne, Switzerland (Dienststelle Gesundheit, Ethikkommission des Kantons Luzern, Schweiz) on May 9^th^, 2012 (Ref. Nr. EK: 12025).

### Well-being

In the written questionnaire headache was assessed using the six-item Headache Impact Test (HIT-6) [6]. A summary score of all six items can range from 36 to 78. A summary score of 49 or less is considered as “headache has no impact on your life,” 50 to 55 is considered as “headache has some impact on your life,” 56 to 59 as “headache has substantial impact on your life” and 60 or more as “headache has a very severe impact on your life.” A binary variable was created by using 56 as the cut-off value. Occurrence of tiredness, lack of energy, lack of concentration and rapid exhaustibility (referred to as exhaustibility) during the four weeks prior to fill in the questionnaire were assessed using a four-point Likert scale with categories “never,” “rare,” “moderate” and “severe.” Binary variables were created by combining answer categories “never” with “rare” and “moderate” with “severe”. Physical well-being was assessed using the dimension “Physical Well-being” from the Kidscreen-52 questionnaire. This dimension includes five questions exploring the level of adolescent’s physical activity, energy and fitness [7, 8]. A binary variable was created by using the mean minus half a standard deviation as the cut-off, which is suggested as the guiding principle according to the official Kidscreen questionnaire handbook. For coherent data presentation, the Kidscreen Well-being was inverted to an ill-being scale by considering a low score as ill-being.

### RF-EMF exposure from fixed site transmitters

Far-field exposure from fixed site transmitters (radio and TV broadcast transmitters and mobile phone base stations, where downlink exposure are included) at home and in school were modelled using a geospatial propagation model based on a comprehensive database of fixed site transmitters and on a three-dimensional topography and building model of the study area [9, 10]. The model was initially developed for the NIR-monitoring project of Central Switzerland, the transmitter data were provided by the environmental offices of the cantons involved. The coordinates of the home and school addresses of the study participants were geocoded from the address using the database of the Swiss Federal Statistical Office. The parents’ and teachers’ questionnaires provided information on the number of floors of the building and the floor location of the residence and of the class room for calculating the height of the residence and of the class room [9, 10]. In order to take into account attenuation by buildings, the following damping factors were applied: 3 dB for outer walls, 5 dB for roofs and 0.6 dB/m in the interior of buildings. The building database that has been used for modelling had no information about very new buildings, therefore a damping factor of 4.6 dB was used when building information was missing [10]. Time weighted average exposure per day for each participant was calculated from the modelled exposure at home (weight: 4/5; 19.2 h) and at school (weight: 1/5; 4.8 h taking into account weekend and holidays). Exposure is expressed in units of the power flux density (μW/m^2^) of the electromagnetic wave.

### Statistical analysis

Three main analyses were performed to investigate possible associations between self-reported general well-being and RF-EMF exposure from fixed site transmitters:

- A mixed-logistic cross-sectional regression analysis of a combined dataset consisting of baseline and follow-up data.
- A cohort analysis including all participants without the target symptom at baseline to investigate whether new onset of a symptom was related to the exposure level at baseline.
- A change analysis including all participants without the target symptom at baseline to investigate whether new onset of a symptom was related to an increase in exposure between baseline and follow-up.

The analyses for the mixed logistic cross-sectional regression analyses (a) and the cohort analyses (b) were based on three exposure categories for all variables: exposure below median (reference), 50^th^ to 75^th^ percentile and the top 25^th^ percentile. In the change analyses (c) we compared study participants with an increase in exposure (>0 μW/m^2^) to the remaining study participants who did not experience an exposure increase between baseline and follow-up (reference).

All models were adjusted for age, sex, nationality, school level (college preparatory high school or high school), physical activity, alcohol consumption and education of parents. In the cohort and change analyses we adjusted for confounders at follow-up. Additionally, all models of the cohort and change analyses (b) were adjusted for change in body height between baseline and follow-up.

Linear regression imputation (14 missing values at baseline and 10 missing values at follow-up for alcohol consumption; 7 missing values at baseline and 6 missing values at follow-up for information on body height) or imputation of a common category (2 missing values at baseline and 1 missing value at follow-up for frequency of physical activity; 60 missing values for educational level of the parents) was used to impute missing values in the confounder variables. Statistical analyses were carried out using STATA version 12.1 (StataCorp, College Station, USA). Figures were made with the software R using version R for Windows 3.0.1.

## Results

439 students (participation rate: 36.8 %) aged 12 to 17 years from 24 schools (participation rate: 19.1 %) from rural and urban areas in Central Switzerland participated in the baseline investigation of the HERMES study. The follow-up investigation was on average 12.5 months after baseline. Mean (SD) age of the study participants at follow-up was 15.0 years (0.79) and mean (SD) body height at follow-up was 167.3 cm (8.5 cm). More than half of the study participants were female (59.8 %) and 109 (25.7 %) attended a college preparatory high school. The majority (80.2 %) had Swiss nationality, whereas 13.9 % had mixed and 5.9 % foreign nationality.

Most of the study participants are physically active for 2 -3 times per week (40.0 %) and don’t drink any alcohol (52.5 %). Highest education of the parents was for 50.6 % the Training school followed by College of higher education (29.9 %).

**Fig. 1:**
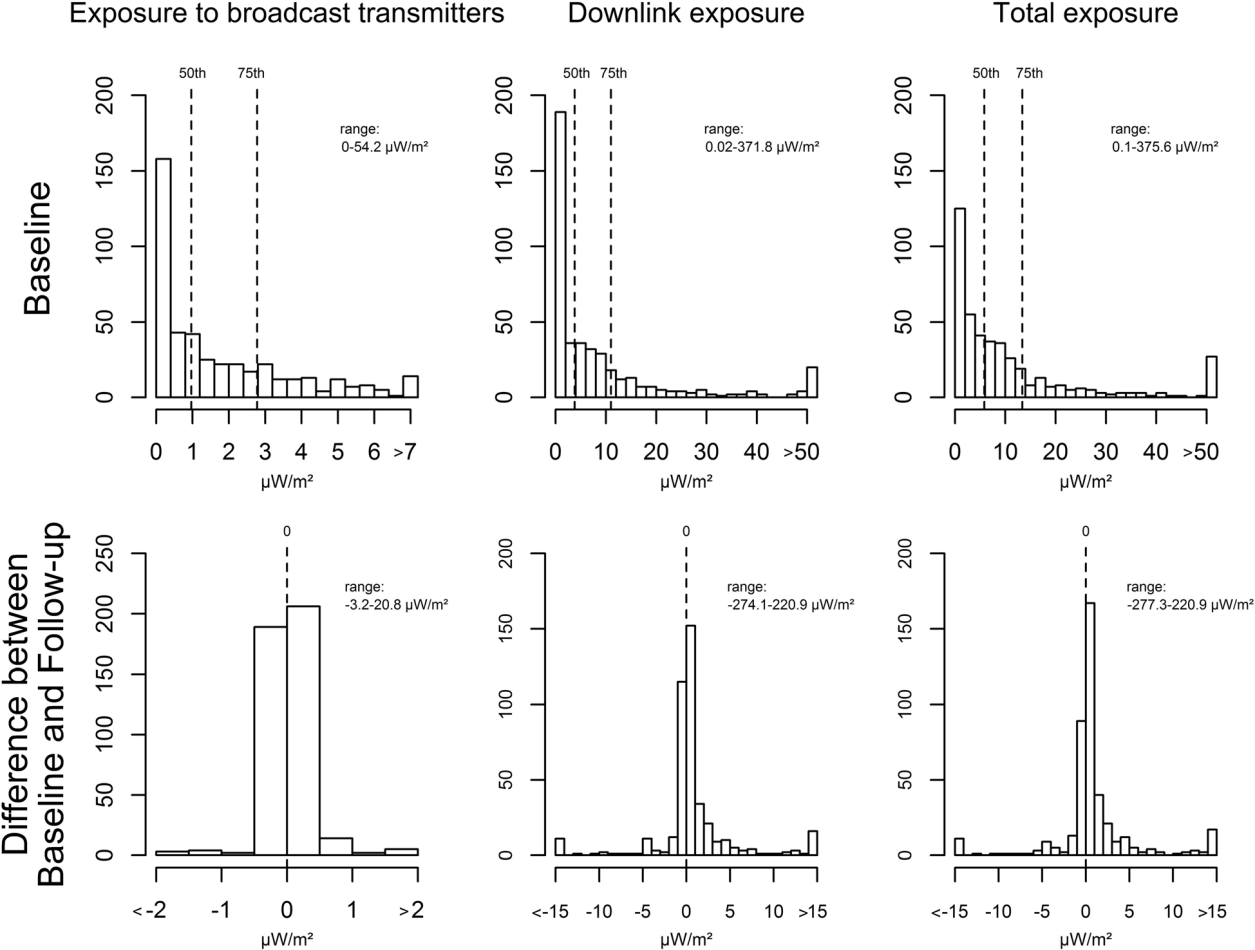
Distribution of the exposure variables

Mean exposure (median; 75^th^) for broadcast transmitters, downlink and total exposure at baseline were 1.9 μW/m^2^ (1.0 μW/m^2^; 2.8 μW/m^2^), 14.4 μW/m^2^ (3.8 μW/m^2^; 11.0 μW/m^2^) and 16.3 μW/m^2^ (5.8 μW/m^2^; 13.4 μW/m^2^), respectively. Mean difference (range) between baseline and follow-up exposure for broadcast transmitters, downlink and total exposure were 0.1 μW/m^2^ (-3.2 to 20.8 μW/m^2^), 0.8 μW/m^2^ (-274.1 to 220.9 μW/m^2^) and 0.9 μW/m^2^ (-277.4 to 220.9 μW/m^2^), respectively. Figure 1 shows the distribution of the exposure variables at baseline with its 50^th^ and 75^th^ percentiles and the distribution of the exposure difference between baseline and follow-up (reference).

### Associations between symptoms and RF-EMF exposure from fixed site transmitters

#### Mixed-logistic cross-sectional analyses (a)

Table 1 shows the results of the mixed-logistic cross-sectional analysis of baseline and follow-up data based on categories. None of the symptoms was significantly associated with any of the exposure measures.

**Table 1:** Odds ratios (OR) of the mixed-logistic cross-sectional analysis of baseline and follow-up data based on exposure categories

	n with symptoms /	Medium exposure (>50^th^ to ≤ 75^th^ percentile)^b^			High exposure (>75^th^ percentile)^b^		
	n total	50^th^ perc [μW/m^2^]	OR (95 % CI) crude	OR (95 % CI) adjusted^a^	75^th^ perc [μW/m^2^]	OR (95 % CI) crude	OR (95 % CI) adjusted^a^
headache							
broadcast transmitter	158/858	0.97	1.23 (0.59 to 2.56)	1.26 (0.60 to 2.63)	2.8	1.79 (0.86 to 3.70)	1.70 (0.82 to 3.54)
total downlink	158/858	4.01	0.72 (0.34 to 1.52)	0.68 (0.32 to 1.45)	11.77	1.21 (0.59 to 2.48)	1.17 (0.57 to 2.38)
total	158/858	6.08	1.23 (0.59 to 2.55)	1.22 (0.58 to 2.55)	14.19	1.29 (0.62 to 2.70)	1.19 (0.57 to 2.49)
tiredness							
broadcast transmitter	404/861	0.97	1.03 (0.59 to 1.81)	1.00 (0.57 to 1.75)	2.8	1.05 (0.60 to 1.86)	1.02 (0.58 to 1.81)
total downlink	404/861	4.01	0.73 (0.42 to 1.27)	0.71 (0.41 to 1.24)	11.77	0.97 (0.56 to 1.69)	0.95 (0.55 to 1.65)
total	404/861	6.08	0.69 (0.40 to 1.21)	0.68 (0.39 to 1.19)	14.19	0.91 (0.52 to 1.59)	0.88 (0.51 to 1.53)
lack of concentration							
broadcast transmitter	163/861	0.97	1.42 (0.73 to 2.76)	1.58 (0.81 to 3.06)	2.8	1.08 (0.54 to 2.15)	1.24 (0.62 to 2.48)
total downlink	163/861	4.01	0.74 (0.37 to 1.44)	0.83 (0.42 to 1.63)	11.77	1.02 (0.52 to 2.01)	1.03 (0.53 to 2.00)
total	163/861	6.08	0.89 (0.46 to 1.74)	1.04 (0.53 to 2.04)	14.19	0.85 (0.43 to 1.69)	0.88 (0.45 to 1.75)
exhaustibility							
broadcast transmitter	131/857	0.97	1.19 (0.66 to 2.14)	1.13 (0.63 to 2.03)	2.8	1.07 (0.59 to 1.94)	0.98 (0.54 to 1.78)
total downlink	131/857	4.01	0.93 (0.52 to 1.66)	0.97 (0.54 to 1.74)	11.77	0.96 (0.54 to 1.74)	0.93 (0.52 to 1.66)
total	131/857	6.08	0.99 (0.56 to 1.77)	1.03 (0.57 to 1.83)	14.19	0.88 (0.48 to 1.60)	0.84 (0.46 to 1.51)
lack of energy							
broadcast transmitter	155/860	0.97	1.19 (0.63 to 2.24)	1.12 (0.60 to 2.11)	2.8	1.09 (0.58 to 2.08)	0.97 (0.51 to 1.86)
total downlink	155/860	4.01	0.70 (0.36 to 1.34)	0.66 (0.34 to 1.27)	11.77	1.10 (0.59 to 2.05)	1.04 (0.56 to 1.94)
total	155/860	6.08	0.90 (0.48 to 1.70)	0.85 (0.45 to 1.63)	14.19	1.13 (0.60 to 2.12)	1.08 (0.58 to 2.01)
physical ill-being							
broadcast transmitter	280/862	0.97	0.95 (0.52 to 1.74)	0.83 (0.46 to 1.48)	2.8	0.93 (0.51 to 1.72)	0.84 (0.47 to 1.50)
total downlink	280/862	4.01	0.97 (0.54 to 1.76)	1.02 (0.58 to 1.81)	11.77	1.39 (0.76 to 2.54)	1.38 (0.78 to 2.45)
total	280/862	6.08	0.83 (0.46 to 1.51)	0.93 (0.52 to 1.64)	14.19	1.20 (0.66 to 2.18)	1.21 (0.68 to 2.13)

^a^ adjusted for age, sex, nationality, school level, physical activity, alcohol and education of parents

^b^ < =50^th^ percentile as reference group

#### Cohort analyses (b)

Table 2 shows the results of the cohort analyses based on categories. Significant associations were found for increased tiredness and high downlink exposure (OR: 3.68; 95%CI: 1.76 to 7.66) and high total exposure to fixed site transmitters (OR: 2.94; 95%CI: 1.43 to 6.05), respectively and for increased lack of concentration and high exposure to broadcast transmitters (OR: 2.78; 95%CI: 1.23 to 6.27). High exposure refers to those in the top 25^th^ percentile compared to those below the median (reference). Further significant results were found for increased lack of concentration for those in the medium broadcast transmitter exposure group (OR: 2.86; 95%CI: 1.28 to 6.42).

**Table 2:** Odds ratios (OR) of the cohort analysis based on exposure categories

	n with symptoms /	Medium exposure (>50^th^ to ≤ 75^th^ percentile)^b^			High exposure (>75^th^ percentile)^b^		
	n total	50 ^th^ perc [μW/m^2^]	OR (95 % CI) crude	OR (95 % CI) adjusted^a^	75 ^th^ perc [μW/m^2^]	OR (95 % CI) crude	OR (95 % CI) adjusted^a^
headache							
broadcast transmitter	40/341	0.96	1.23 (0.55 to 2.73)	1.17 (0.52 to 2.66)	2.8	1.45 (0.65 to 3.25)	1.26 (0.55 to 2.91)
total downlink	40/341	3.79	0.65 (0.26 to 1.58)	0.57 (0.23 to 1.44)	11.01	1.20 (0.56 to 2.57)	1.07 (0.49 to 2.34)
total	40/341	5.82	0.77 (0.32 to 1.81)	0.67 (0.27 to 1.62)	13.38	1.11 (0.51 to 2.43)	0.95 (0.42 to 2.14)
tiredness							
broadcast transmitter	73/228	0.96	0.83 (0.41 to 1.67)	0.67 (0.32 to 1.41)	2.8	1.44 (0.74 to 2.81)	1.35 (0.66 to 2.77)
total downlink	73/228	3.79	1.98 (0.98 to 3.98)	1.71 (0.81 to 3.57)	11.01	3.24 (1.63 to 6.43)	3.68 (1.76 to 7.66)
total	73/228	5.82	1.57 (0.78 to 3.16)	1.47 (0.69 to 3.14)	13.38	2.81 (1.43 to 5.51)	2.94 (1.43 to 6.05)
lack of concentration							
broadcast transmitter	44/343	0.96	2.48 (1.14 to 5.43)	2.86 (1.28 to 6.42)	2.8	2.45 (1.12 to 5.35)	2.78 (1.23 to 6.27)
total downlink	44/343	3.79	1.35 (0.63 to 2.90)	1.51 (0.68 to 3.35)	11.01	1.48 (0.69 to 3.20)	1.51 (0.69 to 3.30)
total	44/343	5.82	1.33 (0.62 to 2.84)	1.64 (0.73 to 3.68)	13.38	1.25 (0.57 to 2.70)	1.31 (0.59 to 2.89)
exhaustibility							
broadcast transmitter	51/361	0.96	1.44 (0.71 to 2.94)	1.28 (0.61 to 2.68)	2.8	1.48 (0.72 to 3.06)	1.32 (0.62 to 2.84)
total downlink	51/361	3.79	1.16 (0.55 to 2.42)	1.00 (0.47 to 2.15)	11.01	1.41 (0.70 to 2.83)	1.33 (0.65 to 2.72)
total	51/361	5.82	1.26 (0.61 to 2.58)	1.08 (0.50 to 2.31)	13.38	1.18 (0.58 to 2.42)	1.10 (0.52 to 2.30)
lack of energy							
broadcast transmitter	53/353	0.96	1.08 (0.52 to 2.24)	1.07 (0.51 to 2.29)	2.8	1.49 (0.74 to 2.98)	1.45 (0.69 to 3.03)
total downlink	53/353	3.79	1.08 (0.52 to 2.25)	1.04 (0.49 to 2.21)	11.01	1.51 (0.76 to 3.00)	1.45 (0.72 to 2.95)
total	53/353	5.82	1.70 (0.84 to 3.41)	1.70 (0.81 to 3.58)	13.38	1.48 (0.72 to 3.03)	1.46 (0.69 to 3.08)
physical ill-being							
broadcast transmitter	55/280	0.96	0.61 (0.28 to 1.34)	0.71 (0.31 to 1.60)	2.8	0.87 (0.42 to 1.80)	0.91 (0.41 to 1.99)
total downlink	55/280	3.79	1.45 (0.70 to 3.00)	1.72 (0.78 to 3.77)	11.01	1.62 (0.80 to 3.27)	1.95 (0.92 to 4.11)
total	55/280	5.82	1.19 (0.57 to 2.47)	1.55 (0.67 to 3.58)	13.38	1.58 (0.78 to 3.19)	2.07 (0.97 to 4.39)

^a^ adjusted for age, sex, nationality, school level, physical activity, alcohol, education of parents and change in body height between baseline and follow-up

^b^ < =50^th^ percentile as reference group

#### Change analyses (c)

In the change analyses two significant results were observed: an increase in downlink exposure was associated with a decrease in lack of concentration and an increase in total exposure to fixed site transmitters was associated with a decrease in exhaustibility (for numbers see Additional file 1: Table S1). None of the symptoms was increased for those with an increase in exposure between baseline and follow-up.

## Discussion

**Fig. 2:**
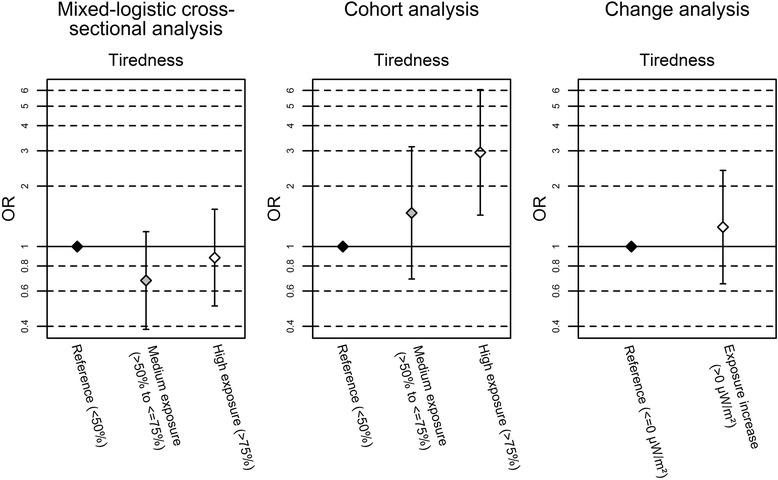
Odds ratios (OR) of the association between tiredness and total exposure to fixed site transmitters. All models are adjusted for age, sex, nationality, school level, physical activity, alcohol and education of parents. The models for the cohort and change analysis are additionally adjusted for change in body height between baseline and follow-up

In cross-sectional analyses of a combined dataset consisting of baseline and follow-up data no associations were observed between any symptom and RF-EMF exposure to fixed site transmitters. In the cohort analyses, where we investigated whether occurrence of the symptom was related to the exposure level at baseline, self-reported tiredness and concentration difficulties tended to be increased in relation to the exposure to fixed site transmitters. But such a pattern was not seen in the cross-sectional and the change analyses (Fig. 2). On the other hand, in the change analyses, where we investigated whether occurrence of symptoms was related to an increase in exposure between baseline and follow-up a decrease of exhaustibility was found for total RF-EMF increase and an improvement in concentration for increase in downlink exposure.

The highest calculated total mean exposure to fixed site transmitters was 375.6 μW/m^2^ (=0.38 V/m), which is considerably below the current ICNIRP (International Commission on Non-Ionizing Radiation Protection [11]) guidelines, as well as lower than the approx. 10 times lower precautionary reference levels in Switzerland as defined by the ordinance relating to protection from non-ionising radiation [12].

A particular strength is the longitudinal design which allows for more robust conclusions compared to cross-sectional studies. To the best of our knowledge, this is the first cohort study on non-specific symptoms in adolescents using a geospatial propagation model to assess exposure from fixed site transmitters. Our model allows prediction of exposure from fixed site transmitters at the homes and at schools of the study participants. We applied different analysis strategies to evaluate varying hypotheses. In order to account for delayed effects with about one year latency (independent of dose relationship), we applied the cohort approach. On the other hand in the change analysis we would find effects if there is a linear relationship and thus we evaluated whether participants with an increase in exposure were more likely to develop symptoms. Thus, results have not to be entirely consistent as different hypotheses are tested but one would not expect to see opposite results as it was the case for us. No longitudinal study with adolescents has been identified so far and only one study in adults was identified to be longitudinal. In this study of 1’124 adults aged between 30 and 60 years no evidence was found that exposure from fixed site transmitters is associated with the development of non-specific symptoms [13] or sleep disturbances [14] over one year.

A further strength is that no information bias can be introduced in the exposure assessment since the exposure is assigned on the basis of residential and school location using a geospatial propagation model and any exposure error is thus not related to the health status. Obviously, there are some uncertainties in the modelling. The uncertainty of these calculations depends on the quality of the input data such as the building and topographic data and the antenna characteristics. A previous validation study for this model in the city of Basel and surroundings found a Spearman correlation coefficient of 0.66 between modelling and indoor measurements conducted in bedrooms during approx. 5 min and a Spearman correlation coefficient of 0.72 between modelling and personal measurements taken during 1 week in the homes of study participants [15]. Additional exposure assessment uncertainty is introduced by the behaviour of the study participants, who do not only stay at home and at school. Exposure outside home and school is not considered in this study.

We are aware that exposure to fixed site transmitters is of minor relevance in comparison to exposure from wireless devices operating close to the body such as a mobile or cordless phone. According to the dose estimations by Roser et al. [16], the far-field exposure from fixed site transmitters contributed on average 0.7 % to the cumulative brain dose and 2.3 % to the cumulative whole body dose. Or expressed differently, the mean dose for the brain in our study sample obtained from mobile phone base stations (downlink exposure) for 24 h corresponds to a mobile phone call of 2.6 s on the GSM (2nd generation Global System for Mobile Communications) network or of a 6.1 min call on the UMTS (3rd generation Universal Mobile Telecommunications System) network. Concerning the exposure to the whole body, 24 h downlink exposure from mobile phone base stations corresponds to a 15.0 s call on the GSM network or to a 34.2 min call on the UMTS network.

However, exposure to fixed site transmitters has different features; the exposure is indeed low, but the levels are more or less constant for several hours a day, especially during night. Further, it is not voluntary and thus not related to lifestyle like wireless device use. Confounding and reverse causality is therefore expected to be less relevant compared to studies focussing on the health effects of mobile phone use.

Nonetheless, we also investigated in our study sample whether self-reported general well-being is associated with a comprehensive RF-EMF brain and whole body dose measure taking into account not only exposure from fixed site transmitters, but exposure from devices operating close to the body such as mobile phones or cordless phones and did not find any indication that symptoms are related to RF-EMF exposure (Schoeni A, Roser K, Röösli M: Symptoms and the use of wireless communication devices: a prospective cohort study in Swiss adolescents, submitted). The absence of associations for these stronger RF-EMF exposure sources calls for a prudent interpretation of the few significant associations observed in our cohort approach. These findings could have happened by chance unless the effect is very frequency or signal specific, for which little evidence is available so far in the low dose range. In particular, the significant association between broadcast transmitters and lack of concentration of the cohort analysis may be due to chance since no exposure response pattern was found. A limitation of the study is the small sample size producing relative large 95 % confidence interval. Our results of the cross-sectional analyses, where we did not find decreased self-reported general well-being in relation to exposure to fixed site transmitters, are in line with other cross-sectional studies on symptoms [2–4].

## Conclusions

Exposure from fixed site transmitters was low in our study area (≤0.38 V/m). In cross-sectional analyses no associations between self-reported symptoms and RF-EMF exposure was observed. In the change analyses a decrease of exhaustibility was found for total RF-EMF increase and an improvement in concentration for increase in downlink exposure, whereas in the cohort approach an association between modelled RF-EMF exposure from fixed site transmitters and tiredness and concentration difficulties in Swiss adolescents was seen. Given the high number of analyses conducted in this study, the observed associations need confirmation before firm conclusions can be drawn.

## Abbreviations

GSM, global system for mobile communications network; HERMES, health effects related to mobile phone use in adolescents; RF-EMF, radiofrequency electromagnetic fields; UMTS, universal mobile telecommunications system network.

## Additional file

Additional file 1:
**Table S1:** Odds ratios (OR) of the change analysis. (DOCX 17 kb)
