# Supplementary material for: Symptoms in Swiss adolescents in relation to exposure from fixed site transmitters: a prospective cohort study
Source: Environ Health. 2016 Jul 16;15:77. doi: 10.1186/s12940-016-0158-4 (PMC4947250; doi:10.1186/s12940-016-0158-4)
Supplement: Additional file 1: — Table S1: Odds ratios (OR) of the change analysis. (DOCX 17 kb) [file 12940_2016_158_MOESM1_ESM.docx]

Table S1. Odds ratios (OR) of the change analysis.

|  | n with symptoms / | n with | exposure increase (> 0 µW/m^2^)** | |
| --- | --- | --- | --- | --- |
|  | n total | exposure increase | OR (95% CI) crude | OR (95% CI) adjusted* |
| **headache** |  |  |  |  |
| broadcast transmitter | 40/341 | 184 | 1.49 (0.75 to 2.93) | 1.35 (0.63 to 2.89) |
| total downlink | 40/341 | 224 | 0.60 (0.31 to 1.17) | 0.60 (0.30 to 1.19) |
| total | 40/341 | 243 | 0.72 (0.36 to 1.44) | 0.73 (0.35 to 1.49) |
| **tiredness** |  |  |  |  |
| broadcast transmitter | 73/228 | 132 | 1.16 (0.66 to 2.04) | 1.09 (0.57 to 2.09) |
| total downlink | 73/228 | 149 | 1.12 (0.62 to 2.02) | 1.16 (0.62 to 2.15) |
| total | 73/228 | 160 | 1.31 (0.70 to 2.45) | 1.25 (0.65 to 2.40) |
| **lack of concentration** |  |  |  |  |
| broadcast transmitter | 44/343 | 184 | 0.85 (0.45 to 1.59) | 0.91 (0.45 to 1.84) |
| total downlink | 44/343 | 219 | **0.47 (0.25 to 0.88)** | **0.46 (0.24 to 0.88)** |
| total | 44/343 | 238 | 0.66 (0.34 to 1.28) | 0.68 (0.35 to 1.32) |
| **exhaustibility** |  |  |  |  |
| broadcast transmitter | 51/361 | 185 | 1.30 (0.72 to 2.36) | 1.39 (0.71 to 2.72) |
| total downlink | 51/361 | 230 | 0.59 (0.33 to 1.08) | 0.55 (0.30 to 1.03) |
| total | 51/361 | 248 | 0.55 (0.30 to 1.00) | **0.50 (0.27 to 0.93)** |
| **lack of energy** |  |  |  |  |
| broadcast transmitter | 53/353 | 181 | 0.90 (0.50 to 1.62) | 1.02 (0.53 to 1.98) |
| total downlink | 53/353 | 231 | 0.77 (0.42 to 1.41) | 0.68 (0.37 to 1.27) |
| total | 53/353 | 247 | 0.60 (0.33 to 1.10) | 0.55 (0.30 to 1.04) |
| **physical ill-being** |  |  |  |  |
| broadcast transmitter | 55/280 | 150 | 0.61 (0.34 to 1.10) | 0.83 (0.42 to 1.64) |
| total downlink | 55/280 | 180 | 1.07 (0.57 to 1.98) | 1.08 (0.56 to 2.10) |
| total | 55/280 | 197 | 0.93 (0.49 to 1.76) | 1.02 (0.51 to 2.02) |

* adjusted for age, sex, nationality, school level, physical activity, alcohol, education of parents and change in height between baseline and follow-up.

** compared to the remaining study participants who did not experience an exposure increase between baseline and follow-up (reference).
